# P076. Improving patient communication and management by the use of the “Headache Digest”, a pilot study

**DOI:** 10.1186/1129-2377-16-S1-A155

**Published:** 2015-09-28

**Authors:** Maria Pia Prudenzano, Paola Mogavero, Eva Moretti, Filomena Puntillo, Paolo Livrea

**Affiliations:** Headache Center, Neurological Clinic “L. Amaducci”, Department of Basic Medical Sciences, Neurosciences and Sense Organs, University of Bari, Bari, Italy; Anaesthesia and Reanimation Clinic, Department of Emergency and Organ Transplants, University of Bari, Bari, Italy

## Background

The average headache visit lasts only 15 minutes in the health care environment and is generally preceded by a long time spent by patients in the waiting room. Patients entering the doctor's room need to be listened to as much as possible but do not know what details might be useful for diagnosis. Doctors focus on obtaining specific clinical information, performing a physical examination, formulating the correct diagnosis and prescribing the most appropriate therapy within as little time as possible. The compilation of a self-administered questionnaire during the waiting time might concentrate a patient's attention on crucial headache features, reduce the tension concerning the visit and break the ice in meeting the doctor. The ID-Migraine, a self-administered questionnaire, consisting of only 3 items, is a valid and reliable screening instrument for migraine and was validated in Italy, but it does not provide information concerning headache course [1].

## Methods

The Headache Digest (HD) is a self-administered questionnaire consisting of 13 items with multiple choice responses constructed according to the ICHD-III beta diagnostic criteria [2]. Seven questions concern headache features. The remaining items collect details regarding onset, clinical course, attack frequency, symptomatic drugs assumption and efficacy. A section is reserved for the doctor dealing with patient's medical history. HD and ID-Migraine were given to 68 consecutive new patients referring to the Bari Headache Center. The completed questionnaires were collected and handed to a designated researcher to analyse data. A headache specialist, blinded to HD and ID-Migraine results, examined each patient and formulated diagnosis according to the ICHD-III beta criteria. HD and ID-Migraine were compared to clinical assessment considered as the reference standard.

## Results

Sixty-two patients received the diagnosis of migraine by the headache specialist. HD showed a sensitivity of 0.98 (95% CI 0.91-0.99) and a specificity of 0.66 (95% CI 0.3-0.90). ID-Migraine showed a sensitivity of 0.75 (95% CI 0.63-0.84) and a specificity of 0.66 (95% CI 0.3-0.90). Seventeen patients (27%) received the diagnosis of migraine with aura by the clinician. HD and clinical evaluation agreed upon headache onset, frequency and symptomatic therapy. Aura was overestimated by HD (44%).

## Discussion

According to this pilot study, Headache Digest is more sensitive than ID-Migraine whereas both tools showed the same specificity. The former also gave clinical details pivotal for diagnosis. The study limitations include small sample size and the aura overestimation. A larger study is in progress based on a revised form of the Headache Digest aimed to improve the diagnostic power of this test.

Written informed consent to publish was obtained from the patient(s).
